# Adrenergic receptor system as a pharmacological target in the treatment of epilepsy (Review)

**DOI:** 10.3892/mi.2024.144

**Published:** 2024-02-27

**Authors:** Ercan Ozdemir

**Affiliations:** Department of Physiology, Faculty of Medicine, Sivas Cumhuriyet University, 58140 Sivas, Turkey

**Keywords:** norepinephrine, adrenergic receptors, α-1 receptors, α-2 receptors, β-receptors, epilepsy, seizure

## Abstract

Epilepsy is a complex and common neurological disorder characterized by spontaneous and recurrent seizures, affecting ~75 million individuals worldwide. Numerous studies have been conducted to develop new pharmacological drugs for the effective treatment of epilepsy. In recent years, numerous experimental and clinical studies have focused on the role of the adrenergic receptor (AR) system in the regulation of epileptogenesis, seizure susceptibility and convulsions. α_1_-ARs (α_1A_, α_1B_ and α_1D_), α_2_-ARs (α_2A_, α_2B_ and α_2C_) and β-ARs (β_1_, β_2_ and β_3_), known to have convulsant or anticonvulsant effects, have been isolated. Norepinephrine (NE), the key endogenous agonist of ARs, is considered to play a crucial role in the pathophysiology of epileptic seizures. However, the effects of NE on different ARs have not been fully elucidated. Although the activation of some AR subtypes produces conflicting results, the activation of α_1_, α_2_ and β receptor subtypes, in particular, produces anticonvulsant effects. The present review focuses on NE and ARs involved in epileptic seizure formation and discusses therapeutic approaches.

## Introduction

Epilepsy is a brain disorder characterized by recurrent seizures, which is diagnosed in 4 to 10 out of every 1,000 individuals in developed countries and affects 75 million individuals worldwide (1-3). The etiology of epileptic disorders is complex and may be of genetic, developmental or acquired origin (4,5). There is a balance between excitatory and inhibitory synaptic mediators [glutamate and gamma-aminobutyric acid (GABA)] in the healthy brain, and a shift of this balance towards excitation is considered the primary cause of epilepsy (6). In addition, serotonergic receptors (7,8), neuroinflammation (9-11), nitric oxide pathway (12) and various ion channels, such as calcium ions (13) may also play a critical role in the mechanism of epilepsy.

There is ample evidence to indicate that the noradrenergic system plays a key role in the regulation of epileptogenesis and convulsions (14,15). Norepinephrine (NE) is generally synthesized and released from noradrenergic nerve endings in the locus coeruleus (LC) (16,17). Abnormal NE secretion causes an increase in tonic/clonic seizures in mice genetically prone to epileptic seizures (18). Although the LC is a small brainstem nucleus, it is the sole source of NE in the neocortex, hippocampus and cerebellum. NE is a potent neuromodulator involved in regulating the excitability of large-scale brain regions. NE concentrations have been reported to increase at seizure onset and decrease during or shortly following the seizure (19).

The inhibition of NE release by gabapentin and pregabalin has an anticonvulsant effect. These drugs exert their effects by binding to the α2δ subunit of voltage-sensitive Ca^2+^ channels. Similarly, gabapentin and pregabalin cause a decrease in NE release through an increase in the extracellular K^+^ concentration (20). In another study, blocking voltage-sensitive Ca^2+^ channels with melatonin exerted an anti-epileptic effect by inhibiting NE release (21). In addition, the density of adrenergic receptors (ARs) in various brain areas decreases during seizures (22,23). NE exerts a pronounced suppressive effect on the development of epileptic seizures. Consistent with this, a decrease in the NE concentration or the administration of AR antagonists causes an increase in the frequency of seizures (24,25). However, there is evidence to suggest that increased NE levels under certain conditions activate seizures, possibly via different ARs (15,26,27). Furthermore, exposure to specific β_2_-adrenergic agonist drugs poses a significant risk for epilepsy (28). Conversely, the β-AR antagonist, propranolol, has been shown to reduce pentylenetetrazole (PTZ)-induced tonic/clonic seizures (29).

The hippocampus plays a crucial role in the pathogenesis of epilepsy and the activation of the α_1A_-AR increases the inhibitory tone in the CA1 region of the hippocampus (30). Selective α_1A_-AR activation increases action potential firing in a subpopulation of hippocampal CA1 interneurons. In response to this, Na^+^ influx is initiated independently of second messenger signaling. In addition, α_1A_-AR activation decreases activity due to increased pre-synaptic GABA in CA1 pyramidal cells (30). Furthermore, blockade of the α_1B_ adrenoceptor subtype exerts both neuroprotective and anti-epileptic effects (31).

The α_2_-adrenoceptor subtype has been reported to modulate seizure susceptibility in different seizure patterns. For example, α_2_-adrenoceptor agonist, clonidine, has been shown to suppress the development of PTZ-induced seizures (32,33). By contrast, the α_2_-adrenoceptor antagonist, yohimbine, has been found to have proconvulsive properties at relatively high doses in the PTZ-induced seizure model (34). Using the α_2_-adrenoceptor pathway, lithium chloride exhibits anticonvulsant properties in the PTZ-induced clonic seizure model (35). Adenosine exerts antiepileptic activity in animals by increasing the seizure threshold induced by PTZ through α_2_-adrenoceptors (36). The β-AR is distributed in the central nervous system (CNS), particularly in the amygdala (37). The decreased expression of β-AR in the amygdala of epileptic animals leads to facilitating seizures (38).

Evidently, the activation of different ARs leads to complex effects on epileptic seizures that have not yet been fully elucidated. In the present review, the role of the adrenergic system in epilepsy and the therapeutic potential of AR agonists are discussed.

## 2. Adrenergic receptor types and subtypes

**Figure 1:**
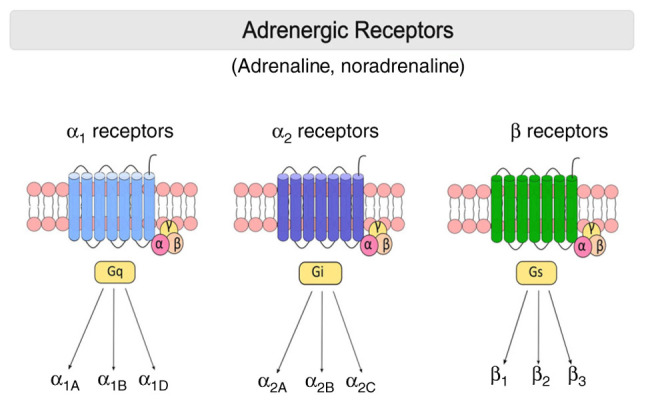
The three adrenoceptor subfamilies and their subtypes. G proteins have a heterotrimeric structure consisting of 3 subunits (α, β and γ). The α subunit can bind guanosine diphosphate and guanosine triphosphate. β and γ subunits mediate the attachment of α to the membrane. α_1_-, α_2_-, and β-ARs mainly couple to Gq, Gi, and Gs proteins, respectively. α_2A_-adrenoceptor subtype agonists often exert their effects by binding to Gi proteins. β-adrenoceptors fundamentally bind to Gs proteins. Gs protein receptors are stimulatory, while Gi proteins are inhibitory.

ARs are membrane-bound G protein-coupled receptors (GPCRs) that mediate the peripheral and central effects of NE. ARs are first divided into two major groups: α- and β-ARs (39). In recent years, the development of new pharmacological tools has revealed nine different subtypes of ARs: Three α_1_-ARs (α_1A_, α_1B_ and α_1D_), three α_2_-ARs (α_2A__/__D_, α_2B_ and α_2C_) and three β-ARs (β_1_, β_2_ and β_3_) (40) (Fig. 1).

In total, three subtypes of α_1_-AR have been identified in the CNS, and α_1A_-ARs are the most abundant (~55%) receptor type. The α_1B_- (35%) and α_1D_ (10%) subtype receptors exhibit a lower distribution (41-43). In particular, α_1_-ARs are abundantly isolated in neurons of the thalamus and cortex, and in interneurons containing GABA (44). α_1A_-AR has a more widespread distribution than α_1B_-AR in the entorhinal cortex and amygdala. Of note, α_1A_-AR is also detected in the cortex, but not in a homogeneous distribution (41). Both α_1_-AR subtypes have been demonstrated in similar cell types, such as neurons, interneurons and progenitors (45,46). Experimental research has demonstrated that α_1A_-AR activation by phenylephrine can significantly reduce hyperexcitability in the hippocampal CA1 region via GABA_A_ receptors (33).

α_2_-ARs have been shown to have both presynaptic and postsynaptic functions. The α_2A_-AR is the main inhibitory presynaptic receptor that regulates NE release from sympathetic neurons as part of a feedback loop (40,47). However, in some tissues, α_2C_-ARs are considered to be inhibitory presynaptic receptors (48). α_2B_-ARs are located on postsynaptic cells and mediate the vasoconstrictive effects of catecholamines released from sympathetic nerves (39).

β-ARs are essential components of the sympathetic nervous system and belong to the superfamily of GPCRs (49). Subsequently, adenylate cyclase (AC) activation causes an increase in cAMP, the main modulator of intracellular events (50). β_1_-AR subtypes constitute 70-80% of cardiac β-ARs (49). β_2_-ARs are mostly found in airway smooth muscle. In addition, β_2_-AR are detected in alveolar type II cells, uterine muscle, mast cells, mucous glands, skeletal muscle, epithelial cells and vascular endothelium (51).

β_3_-ARs are abundantly found in adipose tissue and participate in the regulation of lipolysis and thermogenesis. It has been shown that some β_3_ agonists have anti-stress effects. This suggests that β_3_-ARs also play a role in the CNS. Furthermore, β_3_-ARs have been found in the urinary bladder, gallbladder and brown adipose tissue (52). β_3_-ARs are Gs-type G protein receptors and are involved in norepinephrine-induced AC activation (53).

## 3. Effects of α_1_-adrenergic receptors on epilepsy

Changes in α_1A_-AR intensity have been found in animals with seizures (54,55) and in patients with epilepsy (22). α_1A_-ARs are usually found in postsynaptic neurons and are activated by NE (56). The activation of these receptors specifically inhibits seizures of the limbic system (57). In general, the activation of α-ARs attenuates the rate of epileptiform discharges (58). α_1_-ARs frequently increase the activity of GABAergic interneurons, and GABA released from interneurons plays a key role in the inhibitory effects of these receptors (59,60). By contrast, the overactivity of α_1B_-AR causes spontaneous epileptic seizures in mice overexpressing α_1B_-AR (61), while a deficiency in α_1B_-AR results in the reduction of pilocarpine-induced seizures (31) (Table I) (30,31,62-73).

In the prefrontal cortex, α_1B_-ARs are also expressed in both glutamatergic pyramidal cells and GABAergic interneurons (74). The stimulation of α_1_-ARs depolarizes GABAergic interneurons, resulting in enhanced GABAergic transmission in prefrontal cortex cells (75). In addition, the activation of the α1A-AR subtype by NE also causes the depolarization of hippocampal CA1 interneurons (30). These interneurons are GABAergic and express the neuropeptide somatostatin, and when activated, somatostatin is released to nearby pyramidal neurons. Moreover, the stimulation of α_1A_-AR by NE increases the pre-synaptic release of GABA and somatostatin, thereby reducing CA1 pyramidal activity (76). Furthermore, new pyrrolidin-2-one derivatives with affinity for α_1_-ARs cause a decrease in seizure susceptibility by exhibiting GABAergic activity (77). In addition, it has been shown that seizures originating from the medial prefrontal cortex and caused by acute stress are induced by NE stimulation of α_1_-ARs (65). Electrophysiological recordings have revealed that NE promotes epileptiform activity induction through α_1-_AR stimulation in medial prefrontal cortex pyramidal cells. Similarly, α_1D_-AR antagonism decreases hippocampal glutamate levels and produces potent anticonvulsant effects (78). By contrast, α_1A_-AR stimulation suppresses epileptiform activity in hippocampal interneurons (30).

## 4. Effects of α_2_-adrenergic receptors on epilepsy

**Figure 2:**
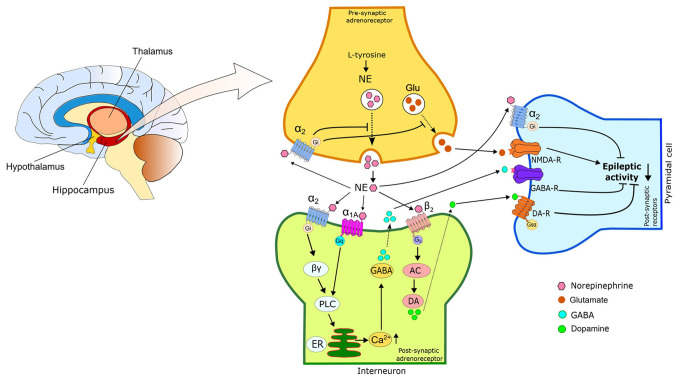
The proposed mechanism of action of the adrenergic receptor system in epileptic seizures. Increased hippocampal NE levels suppress seizures through the activation of α_2A_-AR and β_2_-ARs. Hippocampal NE levels are under negative feedback control of α_2_-ARs. NE controls hippocampal DA, GABA and Glu levels via β_2_-, α_1A_- and α_2_-ARs, respectively. Activation of β_2_-AR by NE increases hippocampal DA levels and suppresses epileptic seizures. Activation of α_1A_-AR and postsynaptic α_2_-ARs increases GABA levels in interneurons and inhibits seizures. Glu secretion by presynaptic α2-AR is suppressed and produces potent anticonvulsant effects. AR, adrenoceptor; NE, norepinephrine DA, dopamine; GABA, gamma-aminobutyric acid; Glu, glutamate; PLC, phospholipase C; ER, endoplasmic reticulum.

α_2A_-ARs are widely distributed in various brain regions, and their activation suppresses the epileptiform activity of areas associated with seizure formation, such as the amygdala (79) and hippocampus (59). Different study data have revealed conflicting results regarding the effects of α_2_ agonists on epileptic seizures. Some data report proconvulsant (27), while others anticonvulsant effects (66,80). In different areas of the brain, α_2A_- and α_2C_-ARs function as both pre- and post-synaptic receptors. It exerts the proconvulsant effects of α_2_-AR agonists through presynaptic α_2_-ARs (81). These agonists reduce NE release in noradrenergic neuron terminals (82). However, the anticonvulsant effect of α_2_-ARs occurs as a result of the released NE activating postsynaptic receptors in target neurons (83). There is also evidence to suggest that post-synaptic α_2A_-receptors are primarily responsible for the anticonvulsant effect of α_2_-adrenoreceptor agonists (59,70). The anticonvulsant mechanism of action of NE is briefly summarized in Fig. 2.

Increasing extracellular hippocampal dopamine and GABA secretions plays a critical role in the anticonvulsant effect of the NE reuptake inhibitor maprotiline. Moreover, the anticonvulsant effect of maprotiline is potentiated by the administration of a selective α_2_- and β_2_-agonists. On the other hand, α_1D_ receptor agonists reduce the anticonvulsant effect (78). The α_2_-AR selective agonist, dexmedetomidine, exerts anticonvulsant effects on PTZ-induced seizures, whereas the α_2_-AR antagonist ATI facilitates epileptic seizures in rats (66). Furthermore, dexmedetomidine significantly reduced the number of c-Fos positive cells in the rat brain (66). However, another study demonstrated a pro-epileptic effect of dexmedetomidine in spike-wave epilepsy in WAG/Rij rats (84). In previous a study on the rat hippocampus, the α2-AR antagonist was implicated in the NE-mediated anti-epileptic effect in the CA3 domain (85). Electrical brain stimulation in the rat hippocampus exerts an inhibitory effect on epileptiform activity via α_1_ and α_2_ ARs (86,87). Moreover, the α_2_-AR agonist, yohimbine, and adenosine provide an additive effect to increase the seizure threshold induced by pentylenetetrazole in mice (36). Experimental evidence has revealed that the specific cannabinoid CB_1_ agonist, ACEA, is involved in its anticonvulsant properties by functionally interacting with α_2_-adrenoceptors in PTZ-induced seizures in mice (32).

The effects of α_2_-AR agonists on epileptic seizure activity vary depending on the dose. Clonidine, an α_2_-AR agonist, exerts anticonvulsant effects at high doses, while it is proconvulsant at low doses (88). The difference in this effect may be partly related to the different signaling pathways initiated by the activation of α_2_-ARs. The dose of α_2A_ agonist used and the adenylate cyclase isoform found in different neurons can determine this effect (89).

## 5. Effects of β-adrenergic receptors on epilepsy

Β-ARs are low affinity receptors for NE and are activated during periods of intense LC activation with a high NE release. The prolonged stimulation of β-ARs leads to a decrease in their sensitivity (90). β-AR is extensively distributed in the amygdala (37). Long-term antidepressant treatment downregulates β-receptors in the amygdala and leads to an increase in epileptic seizures in rats (24). Similarly, reductions in the concentration of β-ARs in the amygdala of epileptic animals may contribute to facilitating seizures (38). The administration of β_2_-AR agonists to mice also causes a reduction in PTZ-induced seizures (82). In addition, the β_2_-agonist, salbutamol, has been shown to exhibit anti-epileptic activity in maximal electroshock-induced seizures in mice (91).

The role of β-ARs in epileptic seizure susceptibility is largely unclear, and there are conflicting findings in different studies. An increase in seizures may be an expected result in studies using β-AR blockers (92). By contrast, there are different studies demonstrating that β-AR antagonists exert anticonvulsant effects in various animal models of seizures (93,94). The non-selective β-AR antagonist, propranolol, exerts an anticonvulsant effect by blocking the sodium channel rather than its hippocampal effects (95). However, it is stated that a similar mechanism is responsible for the anticonvulsant effect of clenbuterol, which is a β_2_-AR agonist (1). Moreover, the stimulation of β_2_-ARs reduces limbic seizures by increasing hippocampal dopamine levels (78). The α-receptor antagonist, phentolamine, selectively reduces anticonvulsant effects, while the β-receptor antagonist, timolol, blocks proconvulsant activity (96). These results suggest that there are different mechanisms in seizure formation in various animal models. Nevertheless, these results clearly indicate that β_2_-AR activation plays a critical role in the anticonvulsant effect of NE.

## 6. Adrenergic modulation of GABA and glutamate

NE exerts excitatory and inhibitory effects on neuronal excitability, depending on receptor subtypes and locations. However, there is evidence to suggest that the dominant effect of NE suppresses excitability in a number of brain regions (83,97). It is a known fact that the pathogenesis of epileptic seizures is associated with the hyperexcitability of brain neurons. Therefore, it is important that NE reduces excitability in its anti-epileptic effect. The effect of NE on neuronal excitability may be via modulation of the conductivity of ion channels or indirectly, usually through GABAergic and glutamatergic transmission (83). Evidence has shown that activating the noradrenergic system facilitates the presynaptic release of GABA (68). In addition, GABA induces NE release by activating GABA_A_ receptors at noradrenergic nerve terminals (98). NE has the ability to alter the excitability of GABAergic cells in certain brain regions (99). For example, the chronic use of certain antidepressant drugs (e.g., citalopram and fluoxetine) that increase NE levels causes the downregulation of ARs and GABA_A_ receptors (100). This regulation may be one of the possible reasons for the proconvulsant effect of chronic antidepressant therapy. The activation of a1-ARs can cause epileptic seizures by increasing GABAergic transmission in various brain limbic regions, including the hippocampus (101), piriform cortex (100) and amygdala (102). The activation of α_1_-ARs through a decrease in potassium conductivity decreases epileptic seizures in the hippocampus by depolarizing inhibitory interneurons (30,101). In a previous study on the medial prefrontal cortex, it was found that the stimulation of α_1_-ARs with phenylephrine facilitated GABAergic transmission to pyramidal neurons (75).

Numerous noradrenergic neurons from the LC make synaptic connections with GABAergic interneurons in the basolateral amygdala. Through the activation of α_1_-ARs, NE depolarizes GABAergic interneurons in the amygdala and increases GABA transmission. This causes the inhibition of pyramidal glutamatergic cells (103). Stress suppresses NE-mediated GABAergic transmission. Therefore, it is suggested that this is a possible mechanism underlying the increase in stress-induced seizure activity (102). A significant association has been found between the decrease in the density of α_2_-ARs in the amygdala of mice and epileptic seizures (64).

There is evidence to suggest strong associations between the adrenergic and glutamatergic systems in the brain. NE secretion also exerts prominent effects on the neuronal excitatory glutamate system (104). NE plays a key role in regulating the sensitivity of specific postsynaptic glutamate receptors (105). It has been stated that ionotropic glutamate receptors play a critical role in the regulation of NE release, and the activation of glutamate receptors reduces NE levels in the rat hippocampus (104). An increase in glutamatergic activity in the entorhinal cortex leads to the induction of seizures. However, the administration of NE blocks seizure activity in this area (105). NE increases epileptiform activity in the hippocampal dentate gyrus (DG) through *N*-methyl-D-aspartate (NMDA) receptor activation (106). A significant downregulation in β_1_-ARs sensitivity in the DG can reduce the stimulating effect of NE and may thus prevent seizures (105). Furthermore, the epileptic seizures observed in transgenic mice overexpressing α_1B_-AR are considered to result from an increased NMDA receptor number via α_1B_-ARs (107).

## 7. Conclusion and future perspectives

There is ample evidence to suggest that the endogenous neuromediator, NE, is involved in the modulation of different types of epileptic seizures. Depending on the activated AR subtype and brain region, NE sometimes has an anti-convulsant and sometimes a convulsant effect. In addition, NE may modulate seizures through affecting various neurotransmitter systems, particularly GABA and glutamate, or voltage-gated Ca^2+^ and/or K^+^ channels. The seizure activity control activity of NE may be impaired in some cases of increased susceptibility to seizures, such as exposure to high levels of NE due to stress. The results of various studies demonstrated that abnormal increases or decreases in NE levels in the brain may cause an impairment in NE-related functions, which may contribute to an increased seizure susceptibility. In conclusion, recent data indicate that the activation of α_1-_, α_2-_ and β_2-_AR subtypes with selective receptor agonists produces anticonvulsant effects in epileptic seizures. Fully elucidating the effects of AR subtypes on epileptic seizures may be an important target for the pharmacological treatment of epilepsy.

## Figures and Tables

**Table I:** Proconvulsant/anticonvulsant activities of adrenergic receptors.

Receptor subtypes	Compound/expression	Mode of action	Proconvulsant/anti-convulsant	Mechanism of action	(Refs.)
α_1A_	Phenylephrine	Agonist	Anti-convulsant	Activation of the α_1A_-AR prompts release of GABA onto CA1 pyramidal cells	(30)
α_1_	Prazosin	Antagonist	Proconvulsant	α_1_ receptor blockade	(62)
α_1B_	Receptor overexpression	-	Proconvulsant	Overexpression of α_1B_-adrenergic receptor in an animal model of epilepsy	(63)
α_1B_	Receptor deficiency	-	Anti-convulsant	α_1B_-adrenergic receptor deficiency in KO mice	(31)
α_1_	Terazosin	Antagonist	Proconvulsant	Adrenergic α_1_ AR blockade in PTZ model epilepsy	(64)
α_1_	Terazosin	Antagonist	Anti-convulsant	It delays seizures caused by acute restraint stress.	(65)
α_2_	Dexmedetomidine	Agonist	Anti-convulsant	Activation of the α_2_-AR in PTZ model epilepsy	(66)
α_2_	Atipamezole	Selective antagonist	Proconvulsant	Prevents post-traumatic epilepsy	(67)
α_2_	6-Fluoronorepinephrine	Agonist	Anti-convulsant	Inhibits epileptiform activity in the rat hippocampal CA3 region	(68)
α_2_	Clonidine	Non-selective agonist	Proconvulsant	Clonidine acts on presynaptic autoreceptors to reduce NE release	(69)
α_2_	Guanfacine	Selective agonist	Anti-convulsant	Guanfacine exerts its anticonvulsant effect on the postsynaptic receptors of NE	(69)
α_2_	Atipamezole	Selective antagonist	Anti-convulsant	Alters CaMKII and suppresses seizures in rats with genetic absence epilepsy (GAERS)	(70)
α_2_	Yohimbine	Antagonist	Anti-convulsant	Enhancement of the pentylenetetrazole-induced seizure threshold in mice	(36)
α_2_	Clonidine	Agonist	Proconvulsant	Inhibited the anticonvulsant effects of N6-cyclohexyl-adenosine	(36)
β	2-Floronoradrenalin (2-FNA)	Selective agonist	Anti-convulsant	Activation of the noradrenergic locus coeruleus system	(71)
β	Propranolol (icv)	Non-selective antagonist	Anti-convulsant	Anticonvulsant effect through central β2-adrenoceptors.	(72)
β	Propranolol (icv)	Non-selective antagonist	Anti-convulsant	Increases the threshold for lidocaine-induced convulsions	(73)

KO, knockout; PTZ, pentylenetetrazole; icv, intracerebroventricular; CaMKII, Ca^2+^/calmodulin dependent protein kinase II; GABA, gamma-aminobutyric acid.

## Data Availability

Not applicable.
